# Exploring the Inhibitory Effects of Fucosylated Chondroitin Sulfate (FCS) Oligosaccharide Isolated from *Stichopus horrens* and the Derivatives on P-Selectin

**DOI:** 10.3390/md23060236

**Published:** 2025-05-30

**Authors:** Caiyi Li, Huifang Sun, Xi Gu, Wen Long, Guangyu Zhu, Xiaolu Wu, Yu Wang, Pengfei Li, Le Sha, Jiali Zhang, Wenwu Sun, Na Gao, Zhili Zuo, Jinhua Zhao

**Affiliations:** 1 School of Pharmaceutical Sciences, South-Central Minzu University, Wuhan 430074, China; lcytianhua@163.com (C.L.); longw2001en@163.com (W.L.); wxl990808@163.com (X.W.); as420556657@163.com (Y.W.); 13708322361@163.com (P.L.); ch1chaqu@163.com (L.S.); zhangjiali20221123@163.com (J.Z.); 2 School of Chemistry and Materials Science, South-Central Minzu University, Wuhan 430074, China; sunhuifang2021@outlook.com; 3 State Key Laboratory of Phytochemistry and Plant Resources in West China, Kunming Institute of Botany, Chinese Academy of Sciences, Kunming 650201, China; guxi@mail.kib.ac.cn (X.G.); zuozhili@mail.kib.ac.cn (Z.Z.); 4 College of Life Sciences, South-Central Minzu University, Wuhan 430074, China; 15347008757@163.com

**Keywords:** *Stichopus horrens*, fucosylated chondroitin sulfate, reductive amination, P-selectin

## Abstract

Unique fucosylated chondroitin sulfate (FCS) extracted from the sea cucumber *Stichopus horrens* was subjected to deacetylation and deaminative depolymerization to generate oligosaccharide fragments containing anTal-diol, which were further purified to obtain the trisaccharide ShFCS-3. Subsequently, the coupling of ShFCS-3 and 4-azidoaniline was achieved by reductive amination. After purification, the main product ShFCS-A1 and by-product ShFCS-A2 were obtained, which were identified as (N-(L-Fuc_2S4S_-α1,3-D-GlcA-β1,3-D-anTalA_4S6S_-1-)-4-azidoaniline) and (4S)-[2-(3-L-Fuc_2S4S_-α1)-D-GlcA-β1]-2,4,5-trihydroxy-5-sulfated-pent-2-enoic-acid) by 1D/2D NMR spectroscopy, respectively. ELISA experiments revealed that ShFCS-A1 exhibited P-selectin inhibition rates of 19.73% ± 9.60% at 1 μM, 96.28% ± 2.37% at 10 μM, and near-complete inhibition (99.92% ± 0.84%) at 100 μM. ShFCS-A2 demonstrated inhibition rates of 8.29% ± 3.00% at 1 μM, 74.02% ± 8.80% at 10 μM, and maximal inhibition approaching 100% at 100 μM. Cellular-level experiments revealed that ShFCS-A1 and ShFCS-A2 inhibited P-selectin binding to HL-60 cells by 92.72% ± 0.85% and 96.97% ± 1.16% at 100 μM, respectively. Molecular docking analysis indicated binding energies of −5.954 kcal/mol for ShFCS-A1 and −6.140 kcal/mol for ShFCS-A2 with P-selectin, confirming their potent inhibitory effects. These findings highlight the therapeutic potential of FCS oligosaccharides as pharmacophores and provide an important foundation for developing novel small-molecule P-selectin inhibitors.

## 1. Introduction

Fucosylated chondroitin sulfate (FCS) is a structurally distinct glycosaminoglycan found in the sea cucumber body wall [1]. FCS exhibits broad pharmacological activities, among which its potent anticoagulant and antithrombotic properties are particularly remarkable [2], primarily attributed to its distinctive chemical architecture. The fundamental structural motif of FCS consists of a disaccharide repeating unit [→4)-β-D-GlcA-(1→3)-β-D-GalNAc-(1→], with α-1,3-linked fucosyl branches attached to the GlcA residues [3].

P-selectin (P-sel) is a pivotal cell adhesion molecule that plays a critical role in inflammatory responses by mediating intercellular interactions to initiate and regulate inflammatory cascades [4]. P-selectin glycoprotein ligand 1 (PSGL-1), the receptor for P-sel, is expressed on leukocytes and platelets. This interaction facilitates key steps in leukocyte recruitment, including rolling, adhesion, and trans-endothelial migration, thereby amplifying local inflammatory signaling cascades [5]. Thus, inhibiting the P-sel/PSGL-1 interaction represents a potential therapeutic strategy to mitigate inflammatory responses. PSGL-1, a mucin-like homodimeric glycoprotein expressed by virtually all subsets of leukocytes, is characterized by the presence of sialyl Lewis X (sLe^X^) glycan modifications [6]. Structural studies reveal that the P-sel/PSGL-1 interaction primarily depends on the N-terminal domain of PSGL-1, where post-translational modifications—particularly tyrosine sulfation and glycosylation (including fucosylation and sLe^X^)—collectively determine high-affinity binding [7,8].

**Figure 1:**
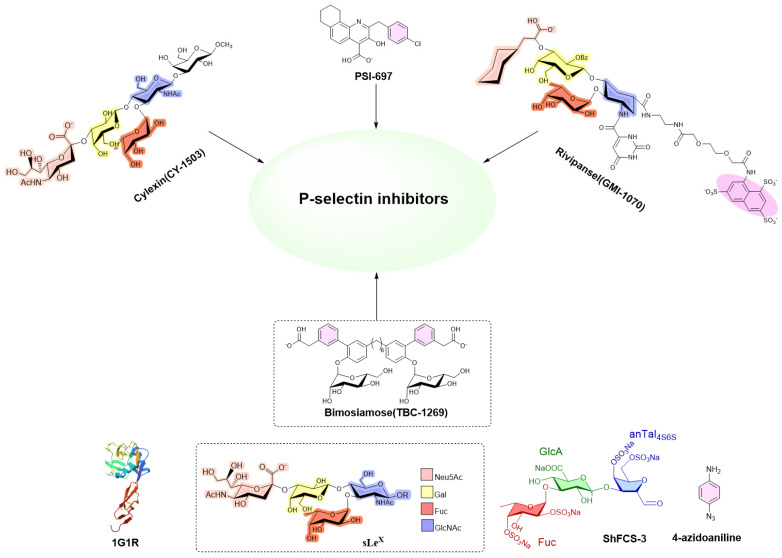
Structures of CY-1503, PSI-697, GMI-1070, sLe^X^, TBC-1269, ShFCS-3, and 4-azidoaniline. ShFCS-3—trisaccharide derived from the sea cucumber *Stichopus horrens*. Fuc (F), fucosyl; GlcA (U), glucuronate; anTalA (T), 1-deoxy-2,5-anhydrate talosyl. P-selectin lectin/EGF domains were complexed with sLe^X^ (PDB: 1G1R).

Given the critical role of P-sel in mediating cellular adhesion, the inhibition or blockade of P-sel activity represents a promising therapeutic strategy for thrombotic disorders. Currently, multiple drug development programs targeting P-sel are in clinical or preclinical stages. P-sel inhibitors primarily fall into three categories: small molecules, antibodies, and glycopeptides. Examples include Cylexin (CY1503) [9], Bimosiamose (TBC-1269) [10,11,12], Rivipansel (GMI-1070) [13,14,15,16], and PSI-697 [17,18,19] (Figure 1); however, these compounds exhibit suboptimal efficacy that fails to meet clinical requirements. Crizanlizumab, the first FDA-approved P-selectin inhibitor for sickle cell disease, is limited by high production costs and poor transport stability [20,21]. Glycopeptides such as GSnP-6, due to their complex synthesis—involving multiple chemical and enzymatic steps—pose significant manufacturing challenges [22,23].

The FCS structural units containing branched fucose residues exhibit spatial conformations analogous to the sLe^X^ oligosaccharide motif in PSGL-1. Studies have demonstrated that FCS derived from *Ludwigothurea grisea* or *Holothuria forskali* can effectively inhibit the sialyl Lewis X (sLe^X^)-mediated interactions between P-selectin/L-selectin and their physiological ligand PSGL-1 [24,25].

In this study, we employed naturally derived fucosylated chondroitin sulfate (FCS) as a structural analogue of sLe^X^. Drawing upon the design concept of the benzene ring in the molecule of TBC-1269, we introduced a benzene ring to enhance the interaction with the binding site. Initially, FCS was extracted from *Stichopus horrens* and subjected to deacetylation and deaminative depolymerization to obtain dShFCS. Subsequent purification via gel permeation chromatography (GPC) yielded a well-defined trisaccharide (ShFCS-3) containing characteristic terminal 2,5-anhydro-D-talose (anTal-diol). We ingeniously introduced a benzene ring structure to enable precise and efficient interaction with the P-selectin binding site, thereby enhancing the inhibitory effect on P-selectin. Through reductive amination, we successfully synthesized the FCS oligosaccharide derivative ShFCS-A1. Interestingly, the by-product ShFCS-A2 also exhibited significant activity. Comprehensive 2D NMR analysis was employed to elucidate their precise structural signals, revealing the chemical structures of ShFCS-A1 and ShFCS-A2. Pharmacological evaluations, including ELISA-based assays, cellular adhesion tests, and molecular docking simulations, demonstrated that these FCS derivatives possess potent P-selectin inhibitory activity. This finding underscores their significant therapeutic potential for treating P-selectin-mediated pathological conditions.

## 2. Results

### 2.1. Extraction, Isolation, and Purification of ShFCS

**Figure 2:**
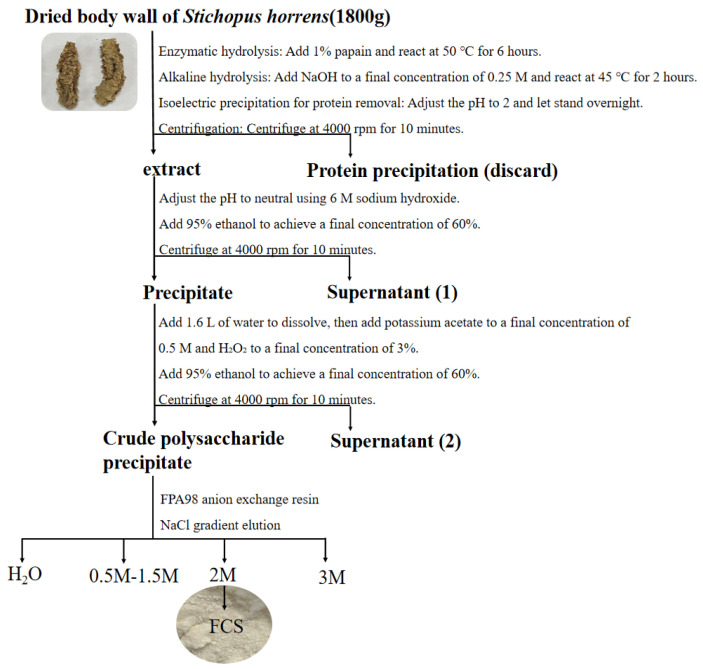
ShFCS extraction from *Stichopus horrens*.

**Figure 3:**
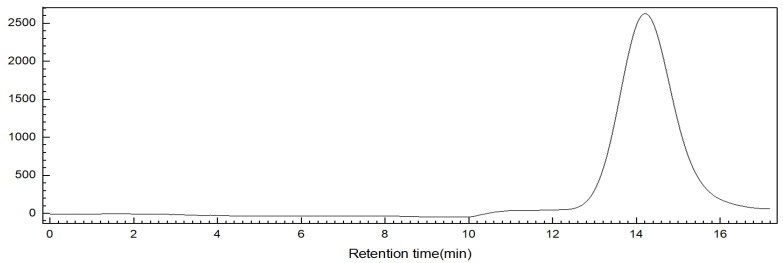
HPGPC of ShFCS from *Stichopus horrens* (Shodex OHpak SB-804 HQ column). HPLC profiles of ShFCS, determined using a Shodex OHpak SB-804 HQ column (8 mm × 300 mm). Aliquots of 50 μL of 2 mg/mL sample were analyzed on an Agilent Technologies 1260 series apparatus equipped with a refractive index (RI) detector eluted with 0.1 M NaCl at a flow rate of 0.5 mL/min.

**Figure 4:**
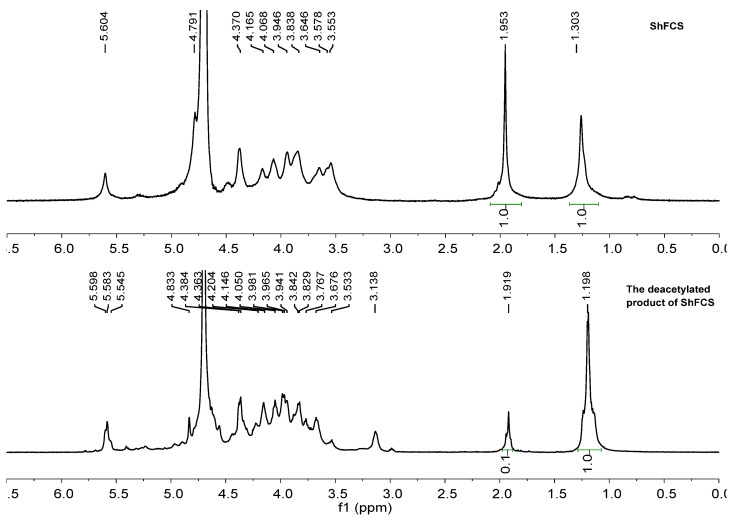
^1^H NMR of ShFCS and deacetylated product of ShFCS.

According to the previous methods for the extraction of FCS from the sea cucumber Stichopus horrens [3] by the method of enzymatic hydrolysis, alkaline treatment, protein removal by isoelectric point precipitation, ethanol precipitation, and purification via FPA98 anion-exchange chromatography, ShFCS was obtained as a white powder (Figure 2). The ShFCS was then analyzed by high-performance gel permeation chromatography (HPGPC) using a Shodex OHpak SB-804 HQ column (Figure 3). The ^1^H NMR signals (Figure 4) of native FCS (ShFCS) from *Stichopus horrens* could be assigned as described previously [26]. In comparison with the FCS structures described in the literature, the signal uniquely detected at 5.604 ppm in this case can be precisely assigned to the anomeric proton of α-L-Fuc_2S4S_. The anomeric proton signals of GlcA and GalNAc fall within the 4.3–4.5 ppm range. Notably, the methyl peaks of GalNAc and Fuc are located at 1.953 ppm and 1.303 ppm, respectively, and their integration ratio is 1:1. ShFCS exhibits a main chain structure composed of repeating units of →4)-β-D-GlcA-(1→3)-β-D-GalNAc-(1→. Moreover, a 2,4-disulfated fucosyl group (α-L-Fuc_2S4S_-(1→) is attached to the O-3 position of the GlcA residue, functioning as a branching structure.

### 2.2. ShFCS Deacetylation

After deacetylation, the deacetylated product of ShFCS was obtained. As shown in Figure 4, the ^1^H NMR spectra of ShFCS and its deacetylated product clearly exhibited the following characteristics: In ShFCS, the -CH_3_ signal of the acetyl group on GalNAc appeared at 1.953 ppm, while the -CH_3_ signal of the side-chain Fuc was located at 1.303 ppm, with an integration ratio of 1:1. In contrast, in the deacetylated product of ShFCS, the integration ratio of the above two -CH_3_ signals was 0.1:1. By comparing the integration ratios before and after deacetylation of ShFCS, the deacetylation degree of ShFCS was determined to be 90%.

### 2.3. Deaminative Cleavage of ShFCS

**Figure 5:**
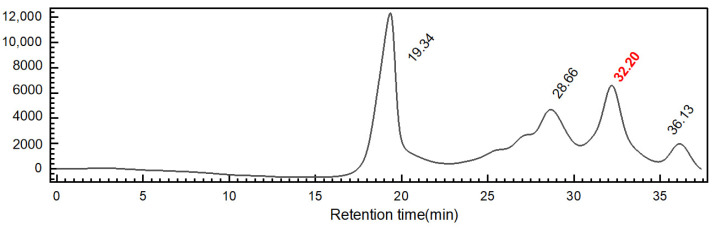
HPGPC of dShFCS (Superdex peptide 10/300 GL). The HPLC profile of ShFCS-3 was analyzed using a Superdex peptide 10/300 GL column (10 mm × 300 mm). A sample of ShFCS-3 (50 μL, 2 mg/mL) was injected and eluted with 0.2 M NaCl at a flow rate of 0.4 mL/min.

The reducing end of the depolymerized product forms a unique structure known as anTal-diol [2]. Based on the known parameters of the FCS depolymerization process, the yield of dShFCS relative to the deacetylated product of ShFCS was 82%. Further analysis on a Superdex peptide 10/300 column showed that it was composed of four fractions with different degrees of polymerization (Figure 5).

### 2.4. Purification of the Oligosaccharide ShFCS-3

**Figure 6:**
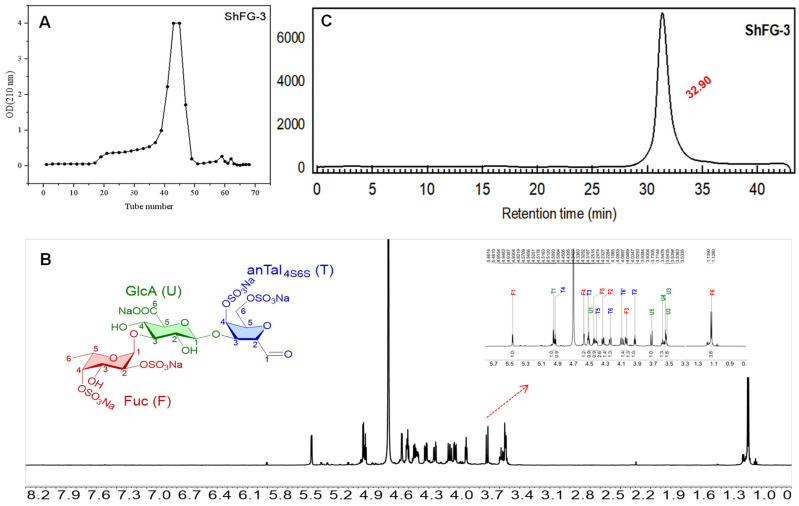
The elution profile of ShFCS-3 (**A**), ^1^H NMR of ShFCS-3 (**B**), HPLC of ShFCS-3 (Superdex peptide 10/300 GL) and Structure of ShFCS-3 (**C**). he HPLC profile of ShFCS-3 was analyzed using a Superdex peptide 10/300 GL column (10 mm × 300 mm). A sample of ShFCS-3 (50 μL, 2 mg/mL) was injected and eluted with 0.2 M NaCl at a flow rate of 0.4 mL/min.

After purification using Bio-Gel P-6 and desalting with Sephadex G-10, the elution profile curve is shown in Figure 6A. The fractionation using GPC with Bio-Gel P6 column combined with analysis using a Superdex peptide 10/300 GL column afforded a range of homogeneous oligosaccharides. By this process, 100 mg of pure trisaccharide (ShFCS-3) was successfully obtained. The yield was 3.6% relative to ShFCS and 14% relative to dShFCS, and its ^1^H NMR spectrum is shown in Figure 6B. Analysis by HPGPC (Superdex peptide 10/300 GL) revealed that the purified ShFCS-3 exhibited a single peak with a purity of >98.0% (peak area normalization method), as shown in Figure 6C. The chemical structure of ShFCS-3 is shown in Figure 6C and primarily consists of three monosaccharides: fucose (Fuc), glucuronic acid (GlcA), and anTal-diol. During the structural analysis of the trisaccharide, the obtained trisaccharide was compared with the data of the trisaccharide in Table 1 of the literature [27]. The analytical results confirmed the structural identity between the two compounds; the H-1 proton of each sugar can be assigned to 5.46, 4.33, and 4.93 ppm for Fuc, GlcA, and 2,5-anhydro-D-talose (anTal-diol), while the small H-H coupling constants (*J*_(1,2)_ = 3.8 Hz) indicate the presence of an α-linkage between Fuc and GlcA, and the larger H-H coupling constants (*J*_(1,2)_ = 8.4 Hz) indicate the presence of a β-linkage between GlcA and anTal-diol. Therefore, ShFCS-3 could be identified as a trisaccharide of L-Fuc_2S4S_-α1,3-D-GlcA-β1,3-D-anTal_4S6S_-diol.

### 2.5. Synthesis, Purification, and Structural Characterization of ShFCS-A1 and ShFCS-A2

We developed a modular synthetic strategy involving three sequential steps—copper-catalyzed azidation, reductive amination, and gel permeation chromatography—to achieve efficient conjugation between ShFCS-3 and 4-azidoaniline. The 4-iodoaniline precursor was first converted to 4-azidoaniline under mild reaction conditions. Site-specific modification of the ShFCS-3 reducing termini was then accomplished through pH-controlled reductive amination. Finally, two major conjugated products were isolated by GPC molecular sieve chromatography.

**Figure 7:**
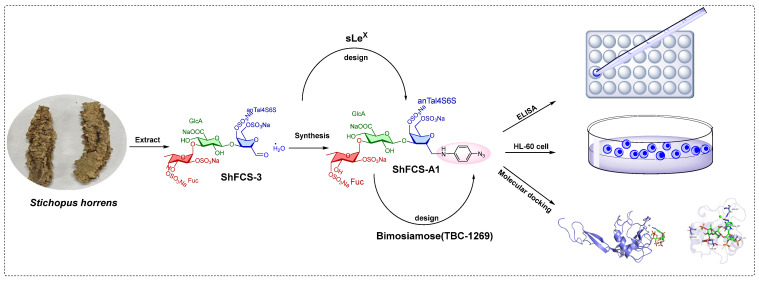
Synthetic strategy for ShFCS-3 derivatives.

**Figure 8:**
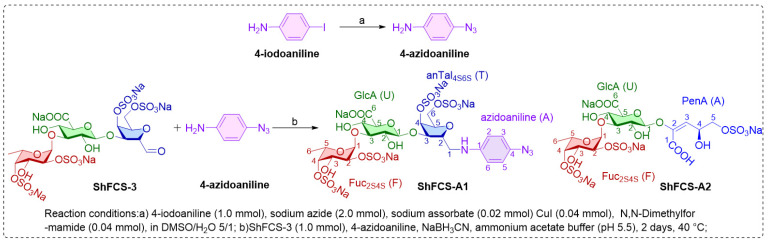
The synthesis route of ShFCS-3 derivatives. ShFCS-A1: N-(L-Fuc2S4S-α1,3-D-GlcA-β1,3-D-anTalA4S6S-1-)-4-azidoaniline; ShFCS-A2: (4S)-[2-(3-L-Fuc2S4S-α1)-D-GlcA-β1]-2,4,5-trihydrox-y-5-sulfated-pent-2-enoic acid; PenA (A), 2,4,5-trihydroxy-5-sulfated-pent-2-enoic acid.

**Figure 9:**
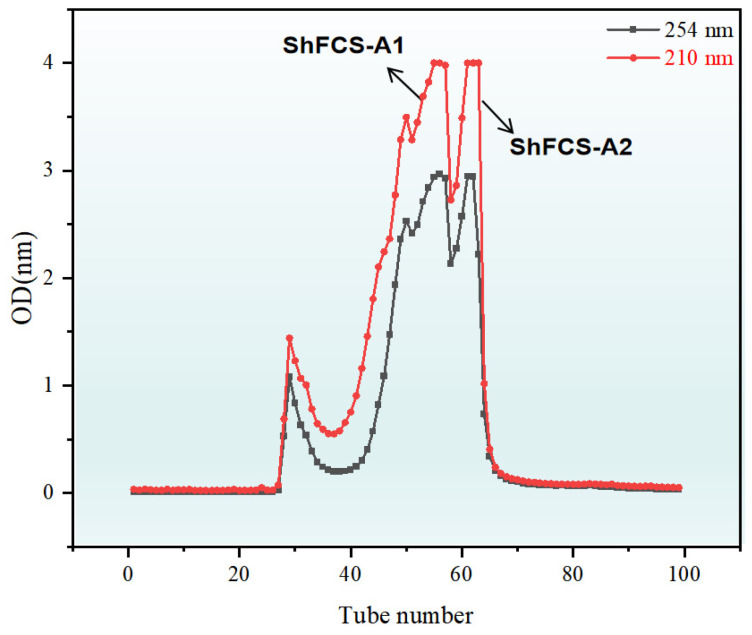
Elution curve of ShFCS-A1 and ShFCS-A2 on Bio-Gel P-2.

The synthetic strategy is shown in Figure 7 and synthesis route in Figure 8. The copper-catalyzed displacement coupling reaction is used to convert 4-iodoaniline to 4-azidoaniline. Subsequently, reductive amination is employed to achieve the coupling of ShFCS-3 as a pharmacophore with 4-azidoaniline. This is followed by purification using Bio-Gel P-2 gel permeation chromatography, which yielded two major products: ShFCS-A1 and ShFCS-A2 (Figure 9). ShFCS-A1 was determined to be N-(L-Fuc_2S4S_-α1,3-D-GlcA-β1,3-D-anTalA_4S6S_-1-)-4-azidoaniline, and ShFCS-A2 was determined to be (4S)-[2-(3-L-Fuc_2S4S_-α1)-D-GlcA-β1]-2,4,5-trihydroxy-5-sulfated-pent-2-enoic acid.

**Figure 10:**
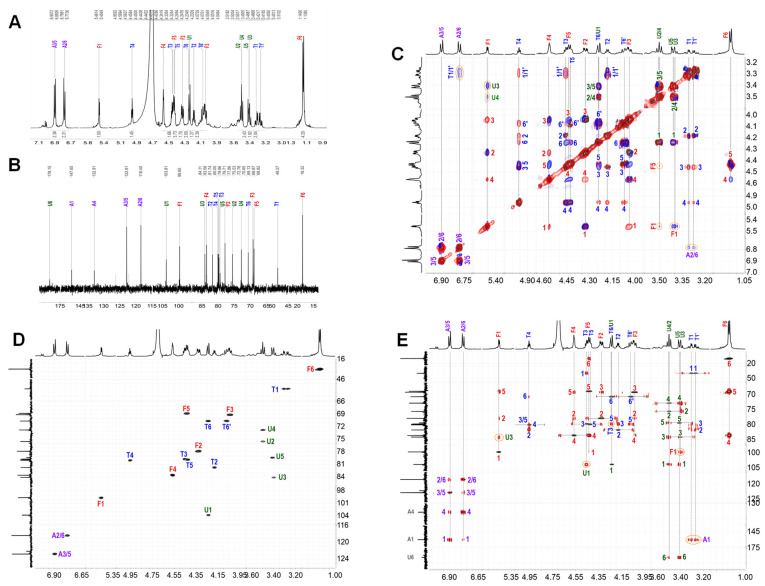
^1^H (**A**); ^13^C (**B**); ^1^H-^1^H COSY (black), TOCSY (red), and ROESY (blue) (**C**); ^1^H-^13^C HSQC (**D**); and ^1^H-^13^C HMBC (**E**) of ShFCS-A1.

**Figure 11:**
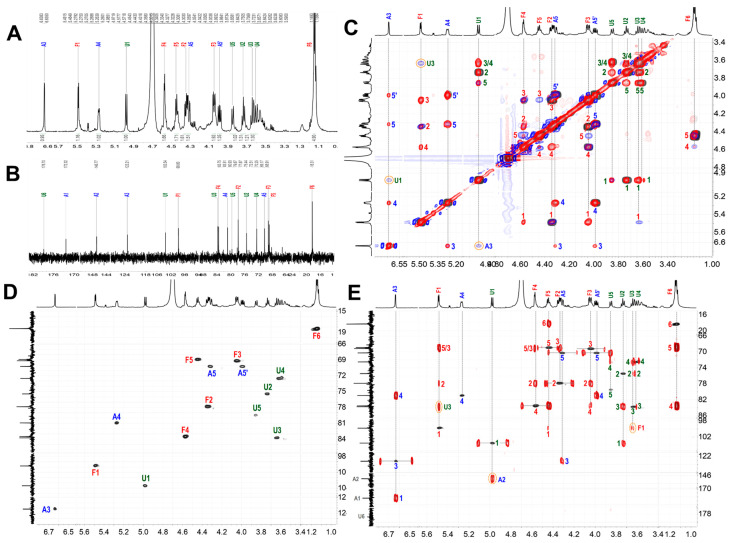
^1^H (**A**); ^13^C (**B**); ^1^H-^1^H COSY (black), TOCSY (red), and ROESY (blue) (**C**); ^1^H-^13^C HSQC (**D**); and ^1^H-^13^C HMBC (**E**) of ShFCS-A2.

The NMR spectra clearly show that ShFCS-A1 contains a trisaccharide (Figure 10A–E). The complete assignment of its peaks is shown in Table 1. The H-1 of each sugar can be assigned to 5.458, 4.241, and 3.319 ppm for Fuc, GlcA, and 2,5-anhydro-D-talitol (anTal-ol), respectively. Their C-1 signals are at 99.65, 103.81, and 48.27 ppm, and azidoaniline can be assigned to 6.780 and 6.900 ppm for A2/6 and A3/5, respectively. Their signals are 118.40 and 112.81 ppm for C2/6 and C3/6. The locations of the attached sulfates on each sugar residue were deduced from the downfield shifts of protons on attached carbons caused by sulfation compared with corresponding unsubstituted monosaccharides, which shows that anTal-ol is sulfated at both the C-4 and C-6 positions and that Fuc is sulfated at both the C-2 and C-4 positions. Additionally, the heteronuclear single quantum correlation (HSQC) (Figure 10D) confirmed these sulfated positions on Fuc and anTal-ol. The small H-H coupling constants (*J*_(1,2)_ = 3.9 Hz) indicate the presence of an α-linkage between Fuc and GlcA, and the larger H-H coupling constants (*J*_(1,2)_ = 7.62 Hz) indicate the presence of a β-linkage between GlcA and anTal-ol. Further analysis using rotating-frame Overhauser effect spectroscopy (ROESY) and heteronuclear multiple bond correlation (HMBC) (Figure 10C,E) confirmed that the C-1 position of Fuc is connected to the C-3 position of GlcA and that a similar (C-1–C-3) connection occurs between GlcA and anTal-ol. From the overlapped ^1^H–^1^H COSY, TOCSY, and ROESY spectra (Figure 11C) and the ^1^H–^13^C HMBC spectrum (Figure 10E), there was related signal connectivity between T1/1′ and A2/6. Additionally, ESI-Q-TOF-MS analysis yielded an *m*/*z* value of 1008.9718 for [M−Na]^−^ (calculated as 1008.9544) (Figure S1). Therefore, ShFCS-A1 is determined to be N-(L-Fuc_2S4S_-α1,3-D-GlcA-β1,3-D-anTalA_4S6S_-1-)-4-azidoaniline.

**Figure 12:**
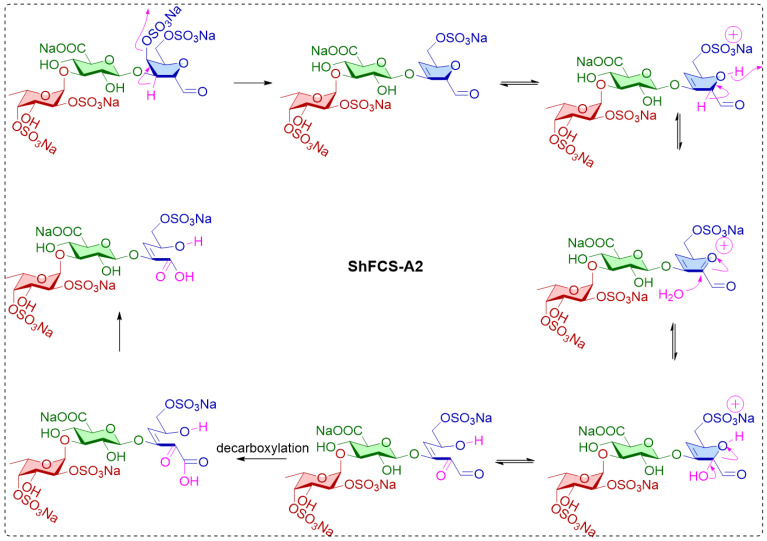
Possible process for the formation of the by-product ShFCS-A2.

Similarly, chemical shifts and sequence assignments for the ShFCS-A2 were achieved as shown in Figure 11A–E and Table 2. Briefly, the signals at 5.448 ppm were H1 of the L-Fuc with α anomeric configurations. The H1 of D-GlcA residue was at 4.979 ppm, and the H1 of the D-GalNAc had β anomeric configurations. From the overlapped ^1^H–^1^H COSY, TOCSY, and ROESY spectra (Figure 11C), there was related signal connectivity between U1 and A3. In the ^1^H–^13^C HMBC spectrum (Figure 11E), there was related signal connectivity between A3 and A1. The upfield shift of A1 at 172.32 ppm indicates it is a carboxyl carbon. In the ^1^H–^13^C HMBC spectrum (Figure 11E), there was related signal connectivity between U1 and A2. The absence of an H signal corresponding to A2 in the HSQC, combined with COSY analysis, indicates the presence of a double bond at this position. In the ^1^H–^13^C HMBC spectrum (Figure 11E), there was related signal connectivity between A3 and A4. Furthermore, the overlapped ^1^H–^1^H COSY, TOCSY, and ROESY spectra confirmed the signal of A5. Additionally, ESI-Q-TOF-MS analysis yielded an *m*/*z* value of 355.9790 for [M-SO_3_Na-Na+H_2_O]^2−^ (calculated as 355.9913) (Figure S2). Therefore, ShFCS-A2 is determined to be (4S)-[2-(3-L-Fuc_2S4S_-α1)-D-GlcA-β1]-2,4,5-trihydroxy-5-sulfated-pent-2-enoic acid. As illustrated in Figure 12, we speculate that the formation of the by-product ShFCS-A2 may occur as follows. Under acidic conditions, the dehydration of threose occurs, opening the ring [28]. Subsequently, the aldehyde group is oxidized to a carboxyl group [29], followed by decarboxylation [30]. The decarboxylated product undergoes further oxidation and finally an elimination reaction to form a double bond [31], leading to the generation of ShFCS-A2.

### 2.6. The Ability of ShFCS-A1 and ShFCS-A2 to Inhibit Binding of P-Selectin to PSGL-1

The higher the binding affinity of the test compounds to P-selectin, the fewer P-selectin molecules are available for subsequent interaction with PSGL-1. P-selectin was immobilized onto the surface of a 96-well plate. Serially diluted sample solutions at varying concentrations were then added to each well. After allowing sufficient binding equilibrium between the samples and the immobilized P-selectin, a PSGL-1-Fc-conjugated anti-human IgG-HRP solution was introduced. The binding affinity of the test compounds to P-selectin is inversely proportional to the binding efficiency of P-selectin to PSGL-1.

**Figure 13:**
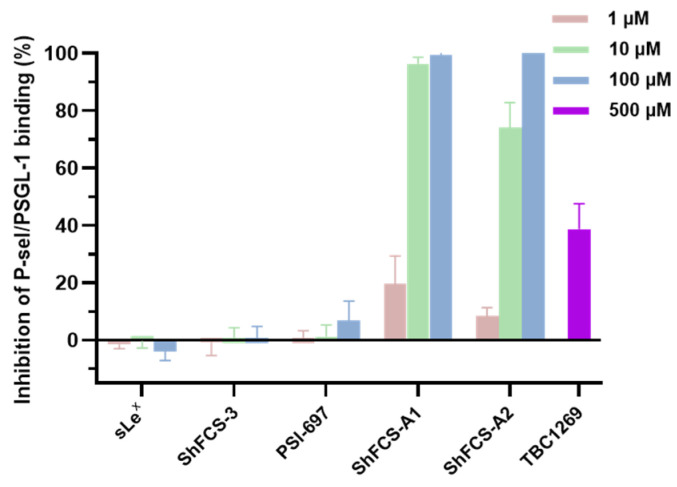
Inhibitory effect of compounds on P-selectin binding to PSGL-1 (*n* = 3).

As shown in Figure 13, ShFCS-A1 and ShFCS-A2 demonstrated superior P-selectin/PSGL-1 binding inhibition compared to sLe^X^ and reference compounds (PSI-697 and TBC-1269).

Specifically, the inhibition rates of ShFCS-3 derivatives at 1 μM were comparable to those of PSI-697 at 100 μM, and at 10 μM, they were comparable to those of TBC-1269 at 500 μM. Specifically, ShFCS-A1 achieved inhibition rates of 19.73 ± 9.60% at 1 μM, 96.28 ± 2.37% at 10 μM, and near-complete inhibition (99.92 ± 0.84%) at 100 μM (*n* = 3). ShFCS-A2 displayed inhibition rates of 8.29 ± 3.00% at 1 μM, 74.02 ± 8.80% at 10 μM, and maximal inhibition (100.50 ± 0.56%) at 100 μM (*n* = 3). These findings highlight the superior potency of ShFCS-A1 and ShFCS-A2 in blocking P-selectin/PSGL-1 interactions compared to sLe^X^ and the reference compound PSI-697, underscoring their potential as effective inhibitors in this context.

Although the experimental sample size was relatively small (*n* = 3), the inhibitory effects observed across different concentration gradients exhibited significant consistency and dose-dependent characteristics, suggesting that the research results have a certain basis for reliability.

**Figure 14:**
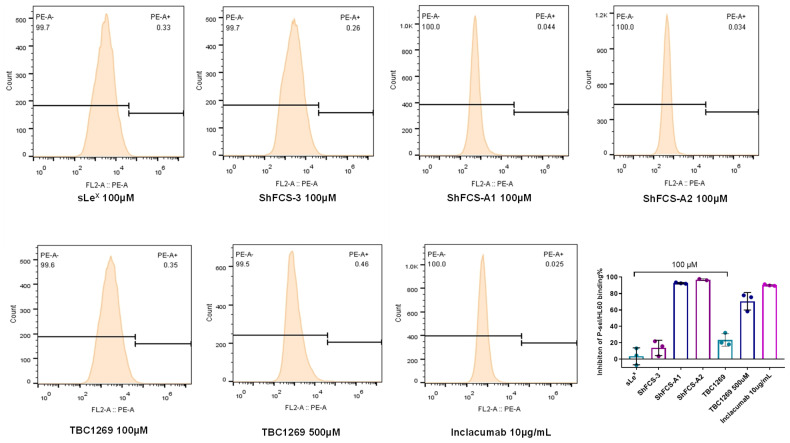
Inhibitory effects of compounds on the binding of P-selectin to HL-60 cells (*n* = 3).

The HL-60 cell line, derived from human promyelocytic leukemia, serves as a crucial model in studies of cell adhesion mechanisms. Under inflammatory or pathological conditions, these cells upregulate PSGL-1, enabling adhesion to P-selectin-expressing cells. This interaction is pivotal in mediating leukocyte–endothelial adhesion during thromboinflammatory responses. The inhibitory effects of ShFCS-A1 and ShFCS-A2 on P-selectin binding to HL-60 cells were assessed using flow cytometry, with results presented in Figure 14. Sialyl Lewis X (sLe^X^) demonstrated minimal inhibitory activity, while non-derivatized ShFCS-3 exhibited an inhibition rate of 13.78 ± 9.30% (*n* = 3) at 100 μM. Notably, at 100 μM, both ShFCS-A1 and ShFCS-A2 showed superior inhibitory activity compared to the positive control TBC1269 at concentrations of 100 μM and 500 μM, with efficacy rivaling that of 10 μg/mL Inclacumab, a P-selectin monoclonal antibody. Specifically, ShFCS-A1 achieved an inhibition rate of 92.72 ± 0.85% (*n* = 3), whereas ShFCS-A2 reached 96.97 ± 1.16% (*n* = 3). These findings underscore the potential of ShFCS-A1 and ShFCS-A2 as effective inhibitors of P-selectin-mediated cell adhesion.

### 2.7. Molecular Docking

**Figure 15:**
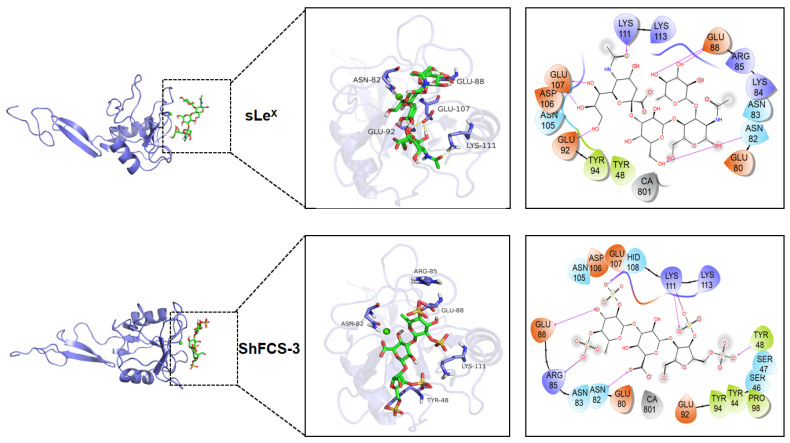

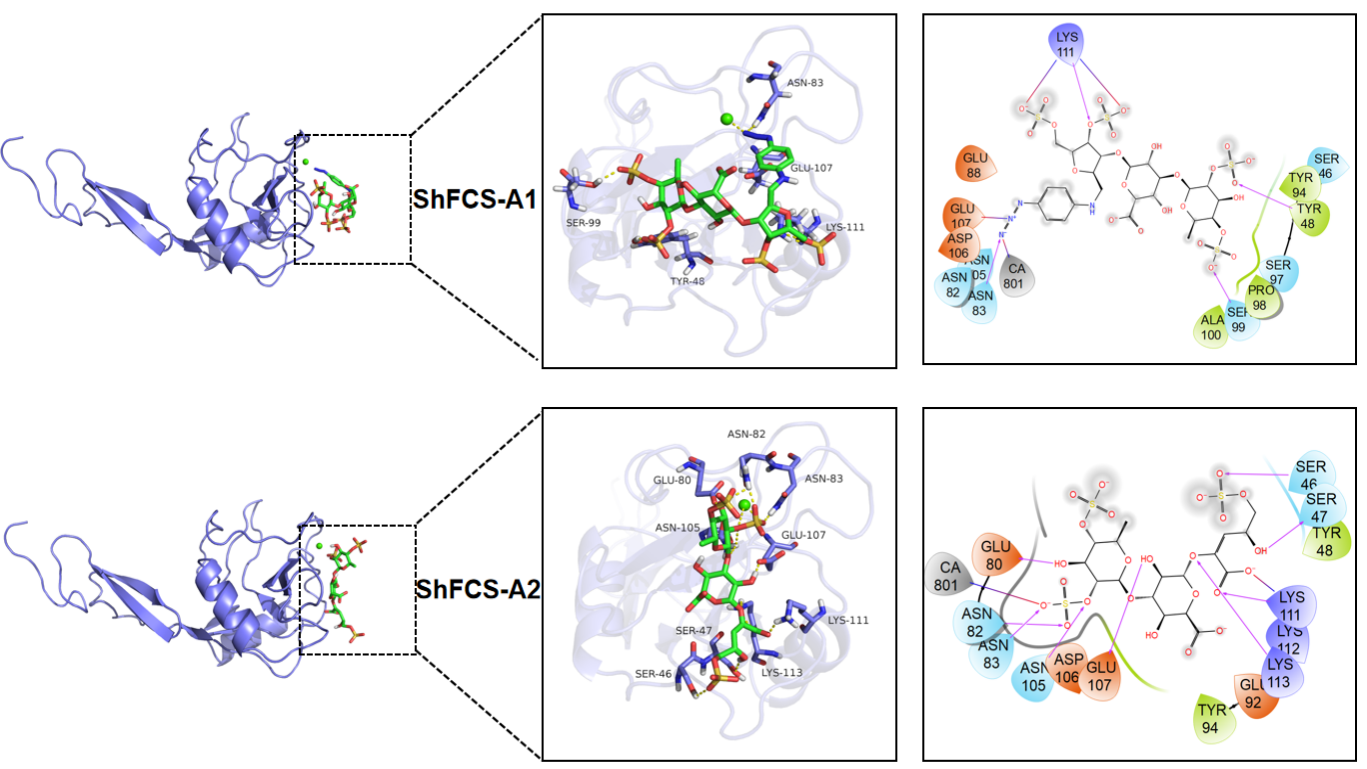
Molecular docking results of sLe^X^, ShFCS-3, ShFCS-A1, and ShFCS-A2.

Molecular docking, a computational chemistry method, predicts the binding mode and affinity between small molecules (ligands) and large molecules (receptors). By analyzing the docking results, we can understand the ligand–receptor interactions, making molecular docking a commonly used auxiliary tool in drug design and molecular function research. As shown in Figure 15, the molecular docking results display the three-dimensional interactions between the compound and P-selectin (1G1R). The greater the absolute value of the binding energy, the stronger the compound’s affinity for 1G1R. The molecular docking values of the compounds with 1G1R are shown in Table 3.

Comparative molecular interaction analysis reveals distinct binding patterns between sLe^X^ and the fucosylated chondroitin sulfate derivatives (ShFCS-3, ShFCS-A1, and ShFCS-A2). While the hydroxyl groups on sLe^X^ form multiple hydrogen bonds with protein residues (Glu88, Asn82, Glu92, and Glu107), its limited anionic character (lacking sulfate groups) results in weaker electrostatic interactions compared to the sulfated derivatives. ShFCS-3 demonstrates a complex interaction network, with its sulfate groups engaging in both electrostatic interactions (Arg85 and Lys111) and hydrogen bonding (Tyr48, HID108, and Lys111), while hydroxyl and carboxyl groups form additional hydrogen bonds (Glu88 and Asn82). The absence of Ca^2+^ coordination may contribute to its relatively lower bioactivity. In contrast, ShFCS-A1 exhibits enhanced binding characteristics through its sulfate groups (electrostatic with Lys111; hydrogen bonds with Tyr48, Ser99, and Lys111) and a unique azide moiety that forms both hydrogen bonds (Asn83) and electrostatic interactions (Glu107 and Ca^2+^), potentially explaining its superior activity. ShFCS-A2 displays the most extensive interaction profile, with sulfate groups participating in hydrogen bonding (Asn82, Asn83, Asn105, and Ser46) and Ca^2+^ coordination, while hydroxyl groups (Glu80, Glu107, and Ser47) and ether oxygen atoms (Lys113) contribute additional hydrogen bonds. Notably, its carboxylate groups establish dual hydrogen bonding and electrostatic interactions with Lys111. The synergistic combination of these electrostatic interactions (involving both sulfate/carboxylate groups and Ca^2+^) and an extensive hydrogen bonding network collectively accounts for the pronounced bioactivity of ShFCS-A2. These structural insights provide a molecular basis for understanding the enhanced P-selectin inhibitory activity of these sulfated derivatives compared to sLe^X^.

## 3. Materials and Methods

### 3.1. Materials and Reagents

Dried body wall of *Stichopus horrens* was purchased from Guangdong, China. Methanol, sodium chloride, sodium hydroxide, ammonium acetate, hydrochloric acid, potassium acetate, concentrated sulfuric acid, 30% hydrogen peroxide, and anhydrous ethanol, all of analytical reagent grade, were purchased from Sinopharm Chemical Reagent Co., Ltd (Beijing, China). Papain was purchased from Beijing Huatai Ruicheng Technology Co., Ltd (Beijing, China). Diacetyl benzidine hydrochloride was purchased from Sigma Company (Burlington, USA). and sodium cyanoborohydride was purchased from Leyan Company (Shanghai, China). pure water was purchased from Wahaha Company (Hangzhou, China). FPA98(OH-) exchange resin was purchased from Amberlit Biotechnology Co., Ltd. (San Francisco, CA, USA). Superdex peptide 10/300 GL, Sephadex G-25 gel, Bio-Gel P-6 gel, and Bio-Gel P-2 gel Bio-Rad were purchased from GE Healthcare (Chicago, IL, USA). 

Tris-HCl was purchased from Solarbio (Beijing China). rhP-selectin (His tag) and recombinant human P-selectin Fc chimeras (P-Fc) were purchased from R&D Systems (Minneapolis, MN, USA). PSI-697 was purchased from MedChemExpress (South Brunswick, NJ, USA). TBC-1269: Mw 862.91, cat HY-106139, lot 59433, MedChemExpress. HRP-Goat Anti-Human IgG was purchased from Polysciences Inc (Warminster, PA, USA). TMB chromogenic substrate (3,3′,5,5′-Tetramethylbenzidine) was purchased from Beyotime Institute of Biotechnology (Jiangsu, China). Pierce™ nickel-coated plates were purchased from Thermo Scientific Company (Waltham, MA, USA). bovine serum albumin (BSA), Tween-20, CaCl₂·2H_2_O, MgCl₂·6H_2_O were purchased from Sigma Company (Burlington, USA). PBS tablets were purchased from MP Biomedicals (Irvine, California, USA). HL-60 cells (human promyelocytic leukemia cells) were purchased from Punosai Life Technology Co., Ltd (Wuhan, China). Lastly, PE-anti-hIgG was purchased from eBioscience (San Diego, CA, USA).

### 3.2. Extraction, Isolation, and Purification of ShFCS-3

#### 3.2.1. Extraction of ShFCS

About 1.8 kg of dried *Stichopus horrens* was soaked in water and then homogenized and digested at 50 °C for 6 h with papain. The solution was cooled to 45 °C and incubated for 2 h after the addition of 6 M NaOH to a final concentration of 0.25 M. The crude polysaccharide was collected as the precipitate by sequential deproteinization, alcohol precipitation (*v*/*v*, 60%), decolorization, alcohol precipitation (*v*/*v*, 60%), and centrifugation (4000 rpm × 10 min) from the extracted mixture. The precipitate was dissolved in water and subjected to anion exchange chromatography on an Amberlite FPA98 column and eluted with H_2_O and 0.5 M, 1.0 M, 1.5 M, 2.0 M, and 3.0 M NaCl solutions. The fraction eluted with 2.0 M NaCl was collected and subjected to desalting using an ultrafiltration membrane. The desalted fraction was determined using an Agilent 1260 infinity II LC system with refractive index detector (RID) on a Shodex OHpak SB-804 HQ column.

#### 3.2.2. Deacetylation and Deamination Depolymerization

ShFCS deacetylation was performed using previously reported method [2]. Briefly, 4.0055 g of ShFCS was placed in a reaction flask with a stirrer, 0.96 g of hydrazine sulfate and 96 mL of hydrazine hydrate were added. The mouth of the tube was sealed with a three-way tube, and N_2_ was introduced for protection. The reaction mixture was heated in an oil bath at 100 °C with stirring at 440 rpm for 45 h. After the reaction, heating and stirring were stopped, and anhydrous ethanol (analytical grade) was added to achieve 80% concentration. The mixture was allowed to stand and then centrifuged at 2500 rpm for 10 min. This process was repeated twice. The final precipitate was dissolved in 32 mL of deionized water, desalted using Sephadex G-25, and lyophilized to yield 2.3 g of the deacetylated product of ShFCS.

The deaminative cleavage protocol was designed according to the literature [32]. The ShFCS deacetylation product was dissolved in 46 mL of deionized water, then 92 mL of freshly prepared 5.5 M NaNO_2_ solution (pH 4.0) was added. The reaction mixture was stirred in an ice bath for 10 min. After the reaction, the pH was adjusted to 7.0 with 1 M NaOH, absolute ethanol was added to 80% (*v*/*v*), and the mixture was allowed to stand. It was then centrifuged at 4000 rpm for 10 min, and the precipitate was redissolved in deionized water, desalted with Sephadex G-25, and the retained fraction was freeze-dried (−50 °C, 0.1 mbar, 48 h) to obtain a white flocculent product (dShFCS).

#### 3.2.3. Isolation and Purification of Oligosaccharide ShFCS-3

dShFCS (300 mg) was dissolved in 5 mL of ultrapure water and subjected to gel filtration chromatography on a Bio-Gel P-6 column (2.5 cm × 180 cm) equilibrated with 0.2 M NaCl. The elution was monitored by UV detection at 210 nm using a BS-100A automated fraction collector (Jiangxi, China), The eluate fraction eluting at a retention time of 32 min, as confirmed by high-performance gel permeation chromatography (HPGPC) using a Superdex™ peptide 10/300 GL column (Marlborough, MA, USA), was collected. The collected fraction was pooled and subjected to desalting via Sephadex G-10 gel filtration. The desalted product was subsequently concentrated via lyophilization, yielding the purified oligosaccharide ShFCS-3.

#### 3.2.4. Nuclear Magnetic Resonance (NMR) Spectroscopy

For nuclear magnetic resonance spectroscopy, 5.0 mg of the sample was dissolved in 0.45 mL of deuterium oxide (D_2_O) and lyophilized. This freeze–dissolve cycle was repeated three times to ensure complete deuterium exchange. The deuterated sample was reconstituted in 0.45 mL of D_2_O and transferred to a 5 mm NMR tube. ^1^H NMR Acquisition: Instrument: Bruker Avance III 600 MHz NMR spectrometer (Karlsruhe, Germany).

Parameters: Spectra were recorded at 25 °C with a spectral width of 20 ppm, an acquisition time of 3.2 s, and 64 scans. Processing: Data were processed using Topspin 4.0 software, with chemical shifts referenced to residual HDO in D_2_O (δ4.70 ppm).

### 3.3. Synthesis and Purification of ShFCS-A1 and ShFCS-A2

#### 3.3.1. Synthesis

According to the method described in the literature [26], 88 mg of 4-iodobenzene (1.0 equivalent) was dissolved in 1.5 mL of DMSO and 0.3 mL of water. Under nitrogen protection, 52 mg of sodium azide (0.05 equivalent), 3.96 mg of sodium ascorbate (2.0 equivalents), 7.62 mg of CuI (0.1 equivalent), and 3.53 mg of N,N-dimethylformamide (0.1 equivalent) were added sequentially. The reaction was monitored by thin-layer chromatography. After the reaction was complete, ethyl acetate and saturated NaCl were added for extraction, and the ethyl acetate layer was collected. Purification via silica gel yielded 42.8 mg of 4-azidoaniline.

According to the method described in the literature [33], the oligosaccharide ShFCS-3 (10 mg, 1.0 equivalent) was added into a 15 mL sealed tube, and 4-(azido)aniline (1.2 equivalents) was added sequentially. This was carried out in the presence of NaBH_3_CN in ammonium acetate buffer (0.15 M, pH 5.5) at 40 °C for 2 days.

#### 3.3.2. Purification

After neutralization, the reaction solution was filtered through a 0.22 μm micropore membrane and purified using Bio-Gel P-2 (2 × 165 cm) gel. The eluent was 0.2 M NaCl, and the elution curve was plotted using UV spectrophotometry at multiple wavelengths of 210/254 nm. Analysis was performed using HPGPC (Superdex peptide 10/30 GL column), and the fractions were merged based on the retention time in 32 min from HPGPC. Fractions with consistent retention times were concentrated, before treatment with Sephadex G-25 (2 × 100 cm) for desalting. The salt-free fractions were concentrated, evaporated under reduced pressure, and lyophilized to obtain the target compounds ShFCS-A1 (3.0 mg) and ShFCS-A2 (2.9 mg).

#### 3.3.3. 1D/2D NMR Analysis

For 1D/2D NMR analysis, 2.5 mg of the sample was accurately weighed and dissolved in 0.45 mL of D_2_O. Lyophilization was carried out, and this operation was repeated three times to ensure complete deuterium exchange. After lyophilization, the sample was dissolved in 0.45 mL of D_2_O and transferred to a 5 mm NMR tube. ^1^H NMR, ^13^C NMR, HMBC, HSQC, COSY, and other related spectra were measured using an Avance III 600 MHz nuclear magnetic resonance spectrometer (Karlsruhe, Germany). Structural analysis of the FCS derivatives: Mestre Nova 11.0 was used for spectral analysis.

### 3.4. Inhibition of P-Selectin Binding to PSGL-1 by Compounds

The competitive binding activities of ShFCS-A1 and ShFCS-A2 with PSGL-1 to P-sel were determined using ELISA. The wash buffer contained 1 mM CaCl_2_, 1 mM MgCl_2_, 0.1% Tween-20, and 1 × PBS. The sample dilution buffer contained 1 mM CaCl_2_, 1 mM MgCl_2_, 1% BSA, 0.01% Tween-20, and 1 × PBS. The experiment included non-specific binding wells, zero concentration sample wells, and wells for the samples to be tested, and was operated as follows: (1) P-selectin coating: First, 100 μL of sample dilution buffer was added to the non-specific binding control wells, and 100 μL of 0.5 μg/mL rhP-selectin (His tag) was added to the remaining wells. The wells were incubated at room temperature for 1 h then washed with 300 μL of wash buffer, repeating the washing process three times. (2) Competition binding of compounds with rhPSGL-1/Fc to P-selectin: First, 100 μL of pre-mixed sample containing different concentrations of the test compound was added to the sample wells (40 μL of 0.75 μg/mL rhPSGL-1/Fc, 40 μL of HRP-anti-hIgG (diluted 1:666), incubated at room temperature for 0.5 h, then 40 μL of the test compound was added at different concentrations). The wells were incubated at room temperature for 1 h then washed with 300 μL of wash buffer, repeating the washing process three times. (3) TMB color development: First, 100 μL of TMB color development solution was added to each well, then the color change in the zero concentration well was observed, and after about 5 min, 100 μL of 2 M sulfuric acid was added to each well to terminate the reaction. (4) Detection: Measure the absorbance value at a wavelength of 450 nm using a microplate reader. (5) Calculation: Calculate the binding rate of the sample, where binding rate = (absorbance value of the sample and control wells − absorbance value of the non-specific binding control well)/(absorbance value of the zero-concentration sample well−absorbance value of the non-specific binding control well) × 100%.

### 3.5. Inhibition of P-Selectin Binding to HL-60 Cells by Compounds

Cultivation of HL-60 Cells: HL-60 cells were purchased from Wuhan Punosai Life Technology Co., Ltd. (Wuhan, China). The cells were cultured using RPMI 1640 medium supplemented with 10% fetal bovine serum (FBS) and 1% penicillin–streptomycin mixture (100 units/mL penicillin, 100 μg/mL streptomycin). The cells were incubated in a 37 °C, 5% CO_2_ incubator, and the medium was changed every two days. Staining solution: PBS containing 1 mM CaCl_2_, 1 mM MgCl_2_, and 0.5% BSA. HL60 cells were washed once with PBS, resuspended in the staining solution, counted, and adjusted to a concentration of 1 × 10^7^ cells/mL. Then, 50 μL of HL-60 cells (each sample tube contains 0.5 × 10^6^ cells) was added to the flow cytometry sample tubes, divided into positive control (without sample), negative control (without P-Fc), and sample groups (100 mM). (1) Adhesion of HL-60 cells to P-Fc: First, 25 μL of 2.5 μg/mL P-Fc was added to the positive and sample groups, and 25 μL of staining solution was added to the negative group. Next, 25 μL of sample was added to each group (25 μL of staining solution for the positive and negative groups), followed by incubation at 37 °C for 30 min. The cells were washed twice with 800 μL of staining solution. (2) PE-anti-hIgG staining: First, 100 μL of PE-anti-hIgG (diluted 1:200) was added to all sample tubes, followed by incubation at room temperature for 30 min. The cells were washed twice with 800 μL of staining solution, resuspended in 600 μL of staining solution, and immediately analyzed on the flow cytometer. The results were analyzed using FlowJo 10.0 software.

### 3.6. Molecular Docking

The X-ray crystal structure (PDB ID: 1G1R) of P-selectin, PSGL-1 peptide, and the polysaccharide complex were obtained from the Protein Data Bank (http://www.rcsb.org/, accessed on 15 May 2025) with a resolution of 1.90 Å [6]. Subsequently, the protein structure was imported into Maestro 12.3 (Schrodinger 2020), and the Protein Preparation Wizard module was used to process the protein complex, adding missing residues and hydrogen atoms, removing water molecules [34]. Additionally, assuming a physiological pH of 7.4, the protonation states of the protein and ligand were adjusted. The protein was then optimized using the OPLS3E force field for further molecular docking studies. For the oligosaccharide structure, the Ligprep module in Maestro 12.3 was used to prepare it under physiological pH 7.4 to generate the corresponding three-dimensional coordinate structures. Docking was performed using the Ligand Docking module, with the receptor target selecting the grid file generated in the previous step and the ligand target selecting the prepared compound structure. The docking precision was set to standard precision [35], and five conformations were generated for each oligosaccharide molecule. The docking results were evaluated based on the docking score (in kcal/mol) and receptor–ligand interactions.

### 3.7. Statistical Analysis

Graphical processing was performed using Origin 2021 and GraphPad Prism 8. All experiments were repeated three times.

## 4. Conclusions

This study successfully extracted fucosylated chondroitin sulfate (FCS) from the sea cucumber *Stichopus horrens*. Through deacetylation–deaminative depolymerization and purification with Bio-Gel P-6, the oligosaccharide ShFCS-3 was obtained. Its purity and structure were determined by liquid chromatography analysis and nuclear magnetic resonance spectroscopy. Using ShFCS-3 as a pharmacophore, ShFCS-3 derivatives were synthesized. 4-azidobenzenami was synthesized from 4-azidoaniline, and then ShFCS-3 was coupled with 4-azidoaniline via reductive amination to generate ShFCS-A1 and the by-product ShFCS-A2. Both were purified with Bio-Gel P-2, and their structures were analyzed by two-dimensional NMR. ShFCS-A1 was identified as N-(L-Fuc_2S4S_-α1,3-D-GlcA-β1,3-D-anTalA_4S6S_-1-)-4-azidoaniline, and ShFCS-A2 as (4S)-[2-(3-L-Fuc_2S4S_-α1)-D-GlcA-β1]-2,4,5-trihydroxy-5-sulfony-l-pent-2-enoic acid, showing their structural differences. Both exhibited good activity in inhibiting P-selectin. Molecular docking simulated their binding to 1G1R, and the results matched those from ELISA and flow cytometry. Given their excellent performance in inhibiting P-selectin, ShFCS-A1 and ShFCS-A2 show great application potential in related fields and offer a new approach for designing P-selectin inhibitors.

However, several scientific questions remain to be addressed. First, although molecular docking and in vitro experiments preliminarily elucidated the binding modes of ShFCS-A1 and ShFCS-A2 to P-selectin (e.g., sulfate-group-mediated ionic interactions and hydrogen-bond networks), the lack of direct validation using biophysical techniques such as surface plasmon resonance (SPR) or isothermal titration calorimetry (ITC) hinders precise quantification of binding affinity (*K*D values) and thermodynamic parameters, thereby limiting the mechanistic depth of the study. Second, the formation mechanism of ShFCS-A2 is currently based on theoretical analysis of NMR data, without experimental validation through real-time monitoring of reaction intermediates via high-resolution mass spectrometry or dynamic profiling of pH-time kinetics, which may compromise the completeness of the proposed reaction pathway. Furthermore, the current derivative library comprises only two structural analogs, lacking systematic structural modifications (e.g., regioselective sulfation or site-specific introduction of alternative pharmacophores), which restricts comprehensive identification of key pharmacophoric features. While ShFCS derivatives exhibit potent P-selectin inhibitory activity in vitro and demonstrate promising potential as anti-tumor and anti-inflammatory therapeutics, their clinical translation faces significant pharmacological challenges, particularly concerning the metabolic stability of sulfate moieties and oral bioavailability. The polyanionic nature of these sulfated polysaccharides renders them susceptible to enzymatic degradation and desulfation in the gastrointestinal tract, potentially compromising their therapeutic efficacy. To address these limitations, several strategic approaches are being pursued: (1) development of prodrug formulations through protective acetylation of sulfate groups, (2) implementation of nanodelivery systems (e.g., chitosan-based nanoparticles) to enhance intestinal absorption, and (3) optimization of intravenous formulations for improved pharmacokinetic profiles. Notably, the incorporated azide functionality not only augments biological activity but also provides a versatile handle for targeted modification via click chemistry, thereby offering unique advantages for subsequent pharmaceutical development and dosage form optimization.

Addressing these limitations could facilitate the rational design of next-generation P-selectin inhibitors, further expanding the therapeutic potential of marine natural products in drug discovery and providing novel treatment options for cardiovascular and cerebrovascular or cancer diseases.

## Figures and Tables

**Table 1:** Signal assignment of 1D/2D NMR for ShFCS-A1.

	^1^H	*δ*	Couplings	COSY	TOCSY	ROESY	^13^C	*δ*	HSQC	HMBC
F	H1	5.458	*J*_(1,2)_ = 3.90	H2	H2,3,4	H2; U2,3	C1	99.65	H1	H5; U3
	H2	4.330	*J*_(2,3)_ = 10.50	H1,3	H1,3,4	H1,3	C2	77.86	H2	H1,2,3,4
	H3	4.041	*J*_(3,4)_ = 3.18	H2,4	H1,2,4	H2,4,5	C3	69.13	H3	H1,2,3,4
	H4	4.566		H3	H1,2,3	H3,5,6	C4	83.58	H4	H3,4,5,6
	H5	4.435	*J*_(5,6)_ = 6.60	H6	H5	H3,4,6	C5	68.82	H5	H1,4,6
	H6	1.143		H5	H6	H4,5	C6	18.32	H6	H5
U	H1	4.241	*J*_(1,2)_ = 7.62	H2	H2,3,4,5	H3,5; T3	C1	103.81	H1	H1,2,5; T3
	H2	3.503	*J*_(2,3)_ = 8.88	H1,3	H1,3,4,5	H1,3; F1	C2	75.53	H2	H2,3,5
	H3	3.401	*J*_(3,4)_ = 9.00	H2,4	H1,2,4,5	H1,2; F1	C3	84.11	H3	H2,3,4; F1
	H4	3.502	*J*_(4,5)_ = 10.02	H3,5	H1,2,3,5	H5; F1	C4	72.78	H4	H3,4,5
	H5	3.413		H4	H1,2,3,4	H1,3	C5	79.39	H5	H4,5
							C6	178.15		H4,5
T	H1	3.319	*J*_(1,2)_ = 4.68	H2	H2,3,4	H2,3; A2/6	C1	48.27	H1/1′	H1/1′,3
	H1′	3.263	*J*_(1,1′)_ = 14.16	H2	H2,3,4	H2; A2/6				
	H2	4.186	*J*_(2,3)_ = 11.46	H1,3	H1,3,4	H1,3,4	C2	81.74	H2	H1/1′,2,4
	H3	4.455	*J*_(3,4)_ = 5.52	H2,4	H1,2,4,6	H1/1′,2,4,6	C3	79.71	H3	H1,2,4; U1
	H4	4.959	*J*_(4,5)_ = 4.80	H3,5	H1,2,3,5,6	H3,5,6	C4	80.05	H4	H3,4,5
	H5	4.428	*J*_(5,6)_ = 2.64	H4,6′	H4,6/6′	H4,6,6′	C5	79.86	H5	H4,5,6,6′
	H6	4.243	*J*_(6,6′)_ = 11.58	H6′	H4,5,6′	H3,5,6′	C6	70.65	H6,6′	H6,6′
	H6′	4.072	*J*_(6′,5)_ = 9.36	H5,6	H4,5,6	H3,5,6				
A	--						C1	147.65		H3/5; T1/1′
	H2/6	6.780	*J*_(2,3)_ = 8.76	H3/5	H3/5	H3/5	C2/6	118.40	H2/6	H3/5,2/6
	H3/5	6.900	*J*_(5,6)_ = 8.76	H2/6	H2/6	H2/6	C3/5	122.81	H3/5	H3/5,2/6
	--	--					C4	132.91		H3/5,2/6

**Table 2:** Signal assignment of 1D/2D NMR for ShFCS-A2.

	^1^H	*δ*	Couplings	COSY	TOCSY	ROESY	^13^C	δ	HSQC	HMBC
F	H1	5.488	*J*_(1,2)_ = 3.96	H2	H2,3,4	H2; U3	C1	99.80	H1	H1,5; U3
	H2	4.346	*J*_(2,3)_ = 10.50	H1,3	H1,3,4	H1,3	C2	77.87	H2	H1,2,3,4
	H3	4.048	*J*_(3,4)_ = 3.36	H2,4	H1,2,4	H2,4,5	C3	69.17	H3	H1,2,3,4
	H4	4.575		H3	H1,2,3,5	H3,5,6	C4	83.51	H4	H3,4,5,6
	H5	4.448	*J*_(5,6)_ = 6.66	H6	H4,6	H3,4,6	C5	68.91	H5	H1,4,5,6
	H6	1.144		H5	H5	H4,5	C6	18.31	H6	H5,6
U	H1	4.979	*J*_(1,2)_ = 7.92	H2	H2,3,4,5	H5; A3	C1	103.54	H1	H1,2
	H2	3.737	*J*_(2,3)_ = 8.28	H1,3	H1,3,4,5	--	C2	75.44	H2	H3
	H3	3.642	*J*_(3,4)_ = 8.82	H2,4	H1,2,4,5	F1	C3	83.75	H3	H2,4; F1
	H4	3.608	*J*_(4,5)_ = 9.36	H3,5	H1,2,3,5	--	C4	72.51	H4	H3,4,5
	H5	3.851		H4	H1,2,3,4	H1	C5	79.67	H5	--
							C6	178.80		--
A	--						C1	172.32	--	H3,5
	--						C2	146.77	--	U1
	H3	6.636	*J*_(3,4)_ = 1.98	H4	H4,5,5′	U1	C3	123.21	H3	H3,5
	H4	5.269	*J*_(4,5)_ = 3.06	H3,5,5′	H3,5,5′	H3,5	C4	80.93	H4	H3,5′
	H5	4.316	*J*_(6,6′)_ = 11.82	H4,6′	H3,4,5′	H4,5′	C6	70.25	H5,5′	H5,5′
	H5′	3.989	*J*_(6′,5)_ = 6.36	H4,6	H3,4,5	H5				

**Table 3:** Molecular docking values of compounds with 1G1R.

Compounds	Docking Score (kcal/mol)
sLe^X^	−5.620
ShFCS-3	−5.066
ShFCS-A1	−5.954
ShFCS-A2	−6.140

## Data Availability

All data generated or analyzed are available from the corresponding author on request.

## Supplementary Materials

The following supporting information can be downloaded at: https://www.mdpi.com/article/10.3390/md23060236/s1, Figure S1: Mass Spectrum of ShFCS-A1, Figure S2: Mass Spectrum of ShFCS-A2.
