# Supplementary material for: Exploring the Inhibitory Effects of Fucosylated Chondroitin Sulfate (FCS) Oligosaccharide Isolated from Stichopus horrens and the Derivatives on P-Selectin
Source: Mar Drugs. 2025 May 30;23(6):236. doi: 10.3390/md23060236 (PMC12193806; doi:10.3390/md23060236)
Supplement: Supplementary file 1 [file marinedrugs-23-00236-s001.zip › marinedrugs-3642147-supplementary.pdf]

Supplementary data

Exploring the Inhibitory Effects of Fucosylated Chondroitin Sulfate (FCS) Oligosaccharide Isolated from *Stichopus horrens* and The Derivatives on P-Selectin

Caiyi Li [1], Huifang Sun [2], Xi Gu[3], Wen Long [1], Guangyu Zhu [4], Xiaolu Wu [1], Yu Wang [1], Pengfei Li [1], Le Sha [1], Jiali Zhang [1], Wenwu Sun [1, *], Na Gao [1, *], Zhili Zuo[3, *], Jinhua Zhao [1, *]

[1] *School of Pharmaceutical Sciences, South-Central Minzu University, Wuhan 430074, China;*

[2] *School of Chemistry and Materials Science, South-Central Minzu University, Wuhan 430074, China;*

[3] *State Key Laboratory of Phytochemistry and Plant Resources in West China, Kunming Institute of Botany, Chinese Academy of Sciences, Kunming 650201, China;*

[4] *College of Life Sciences, South-Central Minzu University, Wuhan 430074, China;*

* Corresponding author.

E-mail addresses: wenwusun@scuec.edu.cn (W.S.); gn2008.happy@163.com (N.G.); zuozhili@mail.kib.ac.cn (Z.Z.); zhaojhscu@163.com (J.Z.)

ShFCS-A1：$[M-Na]^-$:1008.9718

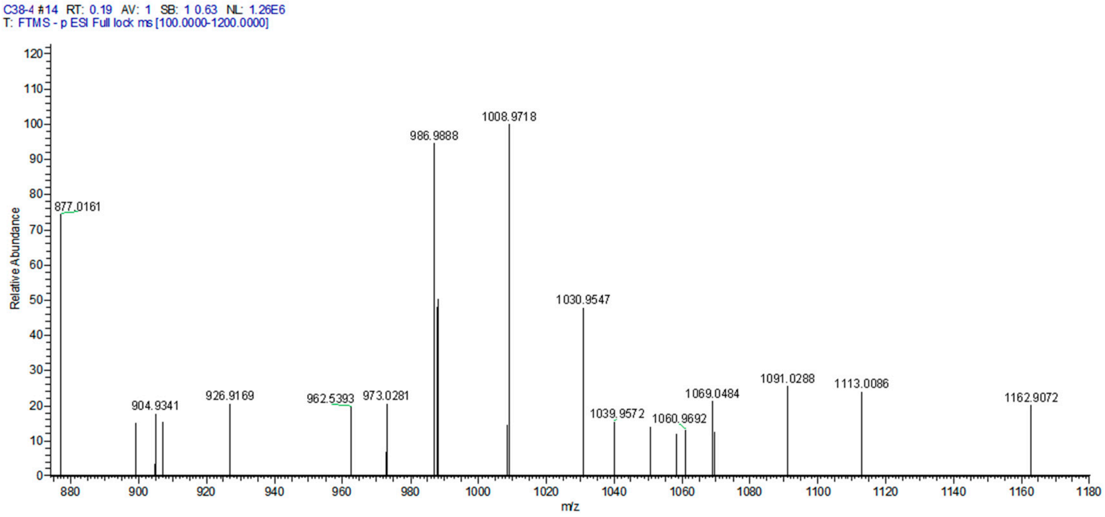

Figure S1. Mass Spectrum of ShFCS-A1

ShFCS-A2： $[M\text{-}SO_3Na\text{-}Na+H_2O]^{2-}$:355.9790

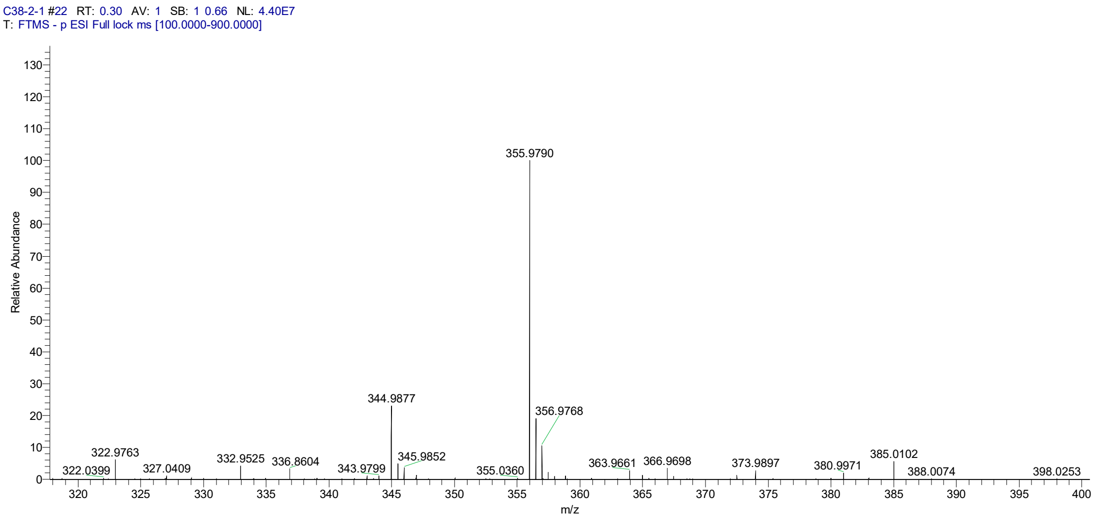

Figure S2. Mass Spectrum of ShFCS-A2
